# Metal and Carbon Support Structure Design Strategies for High-Performance Platinum-Based Hydrogen Evolution Reaction Electrocatalysts

**DOI:** 10.3390/nano16120769

**Published:** 2026-06-18

**Authors:** Seo Jeong Yoon, In-Yup Jeon

**Affiliations:** Department of Chemical Engineering, Wonkwang University, 460 Iksandae-ro, Iksan 54538, Republic of Korea

**Keywords:** hydrogen evolution reaction, metal, carbon support, electrocatalysis, design

## Abstract

Hydrogen (H_2_) has emerged as a promising next-generation energy carrier with significant potential to mitigate climate change and environmental pollution. The hydrogen evolution reaction (HER) is the critical half-reaction directly responsible for hydrogen production. Efficient HER electrocatalysts must exhibit low overpotential values and fast reaction kinetics to achieve high catalytic performance. While platinum (Pt) remains the benchmark catalyst due to its ideal hydrogen adsorption energy, high electrical conductivity, and superior chemical stability, further innovations are essential. This review summarizes recent advances in Pt-based HER catalysts, focusing on two primary design strategies: metal-level engineering and support-level engineering. These approaches allow for precise control over electronic structures, active site distributions, and interfacial properties, paving the way for next-generation HER electrocatalysts.

## 1. Introduction

Hydrogen (H_2_) has emerged as a promising next-generation energy carrier with significant potential to mitigate climate change and environmental pollution. However, conventional hydrogen production processes are predominantly dependent on fossil fuels, leading to considerable CO_2_ emissions and associated environmental concerns [1,2]. In this context, renewable-energy-driven water electrolysis has been widely recognized as a sustainable and environmentally friendly route for high-purity green hydrogen production. During water electrolysis, the oxygen evolution reaction (OER) takes place at the anode, whereas the hydrogen evolution reaction (HER) occurs at the cathode. Among these, HER is the key half-reaction directly responsible for hydrogen generation [3]. Efficient HER electrocatalysts are required to exhibit low overpotential levels and fast reaction kinetics, reflecting both favorable thermodynamics and minimized kinetic barriers [4,5].

Platinum group metals (PGMs), including Pt, Ru, Ir, Rh, and Pd, generally demonstrate outstanding catalytic performance for water-splitting reactions. Among them, Pt serves as the benchmark catalyst for the HER due to its favorable hydrogen adsorption energy, excellent electrical conductivity, and outstanding chemical stability [6]. However, several critical limitations continue to hinder the practical application of Pt despite its significant advantages. These include its high cost, limited natural abundance, low atomic utilization efficiency, and a reduction in the active surface area due to nanoparticle agglomeration [7,8,9]. These challenges pose significant barriers to large-scale production and long-term durability. Therefore, it is essential to develop rational strategies to minimize Pt loading rates while maximizing the atomic utilization efficiency [3]. Accordingly, extensive research efforts have focused on developing advanced catalyst design strategies to minimize Pt usage while maintaining or even enhancing catalytic performance capabilities.

In this review, we summarize recent advances in Pt-based HER catalysts, with an emphasis on two major design directions: metal-level engineering and support-level engineering. These approaches collectively enable fine regulation of the electronic structure, the dispersion of active sites, and control over the interfacial properties, offering a promising pathway toward next-generation HER electrocatalysts.

## 2. Fundamentals of the Hydrogen Evolution Reaction (HER)

### 2.1. HER Mechanism

The hydrogen evolution reaction (HER), which serves as the cathodic half-reaction of electrochemical water splitting, has been one of the most extensively studied reactions in relation to electrocatalysis [10]. As summarized in Table 1, the overall reaction proceeds through two principal mechanistic pathways, the Volmer-Heyrovsky and the Volmer-Tafel mechanisms, which share a common initial step involving the adsorption of atomic hydrogen (H*) onto the catalyst surface [11,12].

In acidic media, H* is generated via the electrochemical reduction of protons (H^+^ or H_3_O^+^) at the electrode surface (Volmer step). The subsequent desorption of H* to form molecular hydrogen can proceed via either the Tafel step, where two adjacent H* species recombine, or the Heyrovsky step, where H* reacts directly with a proton and an electron. The operative pathway depends primarily on the hydrogen binding energy and the electronic structure of the catalyst surface.

In alkaline media, the mechanistic pathway is fundamentally analogous, proceeding through the same Volmer-Heyrovsky or Volmer-Tafel sequences. However, the absence of free protons necessitates an initial water-dissociation step (H_2_O + e^−^ → H* + OH^−^) to generate the H* intermediate prior to the Heyrovsky or Tafel steps, introducing an additional energy barrier. As a consequence, alkaline HER kinetics can generally be two to three orders of magnitude slower than those observed under acidic conditions. The rate-determining step (RDS) is typically inferred from the experimentally measured Tafel slope, with values of approximately 120, 40, and 30 mV dec^−1^ corresponding to the Volmer, Heyrovsky, and Tafel steps, respectively [13]. Together with the overpotential and exchange current density, the Tafel slope constitutes a key kinetic metric for benchmarking electrocatalyst performance outcomes. This mechanistic understanding motivates the use of thermodynamic and electronic structure descriptors as rational design criteria for HER electrocatalysts.

### 2.2. Theoretical Framework for the HER

#### 2.2.1. Hydrogen Adsorption Free Energy (ΔG_H*_)

**Figure 1:**
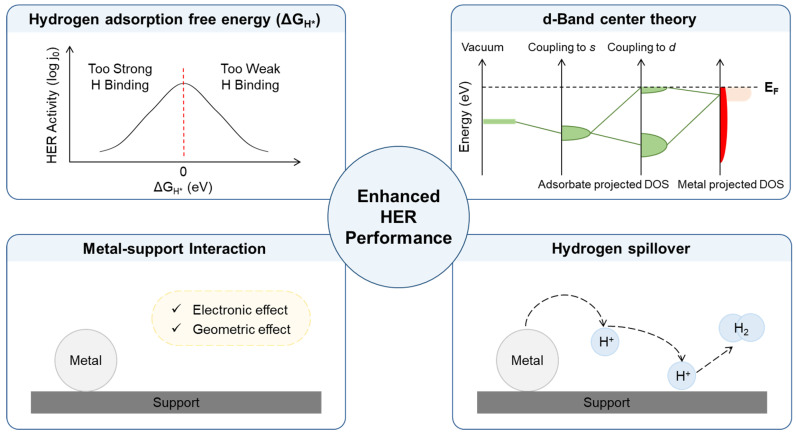
Overview of the fundamental catalytic factors associated with enhanced HER activity.

The hydrogen adsorption free energy (ΔG_H*_) is commonly regarded as the key thermodynamic descriptor for HER activity, grounded in the Sabatier principle and visualized through a volcano plot [14]. Here, we elaborate on the quantitative framework underlying this descriptor and its implications for catalyst design (Figure 1).

ΔG_H*_ is defined within the computational hydrogen electrode (CHE) model, in which the chemical potential of a proton-electron pair (H^+^ + e^−^) is set equal to that of 1/2H_2_ under standard conditions (U = 0 V vs. RHE, pH = 0, T = 298 K) [14]. This approach enables a direct comparison of ΔG_H*_ across diverse catalyst surfaces from DFT calculations without requiring explicit treatment of the electrochemical interface [15].

It should be noted that while ΔG_H*_ ≈ 0 is a necessary condition for high HER activity, it is not always sufficient [16]. In alkaline media, the Volmer step involves water dissociation rather than proton adsorption, introducing an additional energy barrier, as noted above, that is not captured by ΔG_H*_ alone [17]. Accordingly, additional descriptors such as the hydroxyl binding energy (ΔG_OH*_) and the water-dissociation barrier have been proposed as complementary metrics for evaluating alkaline HER activity [18]. Pt exhibits near-thermoneutral ΔG_H*_ levels, yet further optimization through electronic structure engineering is essential to enhance the activity and reduce the Pt loading level.

#### 2.2.2. d-Band Center Theory

Beyond ΔG_H*_ as a thermodynamic descriptor, the d-band center (ε_d_) provides a complementary electronic structure framework for rationalizing the catalytic behavior of transition-metal electrocatalysts [19]. The fundamental stages of electrocatalysis inherently involve an electron transfer between transition-metal reactive species and the catalyst surface. In this regard, ε_d_ governs the strength of these interactions by determining the degree of overlap between metal d-states and adsorbate frontier orbitals.

By precisely adjusting the spatial configuration and energetic distribution of valence electrons on the catalyst surface, one can directly modulate activation energy barriers and thereby influence both the reaction reactivity and selectivity [19]. Based on d-band theory, an upshift of ε_d_ toward the Fermi level strengthens the adsorption of hydrogen intermediates on the metal surface. Conversely, a downshift of ε_d_ weakens this interaction and facilitates hydrogen desorption. Consequently, ε_d_ serves as a key quantitative descriptor for optimizing ΔG_H*_ in HER electrocatalysts. Modulation of ε_d_ has emerged as a primary approach to shifting ΔG_H*_ toward the thermoneutral optimum [20].

#### 2.2.3. Metal-Support Interaction

The interaction between metal nanoparticles and their supports is a central determinant of the catalytic activity, selectivity, and stability of HER electrocatalysis. Metal-support interaction (MSI) encompasses various chemical and physical interactions at the metal-support interface, including the charge transfer, interfacial structural reconstruction, nanoparticle morphology regulation, and chemical composition modulation [21,22,23].

MSI influences the HER catalytic performance through two distinct but synergistic mechanisms. The electronic effect refers to interfacial charge transfer between the metal and support, which modifies the electron density of metal active sites and shifts the d-band center, thereby tuning the hydrogen adsorption free energy (ΔG_H*_) toward the thermoneutral optimum as visualized on a volcano plot. The geometric effect refers to the ability of the support to regulate the nanoparticle size, morphology, dispersion, and exposed facets, thereby controlling the number and nature of accessible active sites and influencing the atomic utilization efficiency. In practice, these two effects operate simultaneously and are difficult to decouple, but their relative contributions depend strongly on the nature of the support material. The synergy between electronic and geometric effects is particularly important for HER catalysis, as electronic effects modulate ΔG_H*_ while geometric effects maximize the exposure and accessibility of active sites. Beyond these two effects, MSI also facilitates hydrogen spillover, where activated H* migrates from metal active sites onto the support surface, providing additional HER reaction pathways. Overall, the rational engineering of MSI represents a versatile and effective approach to optimizing ΔG_H*_, maximizing the active site density, and enhancing catalyst durability for HER applications.

#### 2.2.4. Hydrogen Spillover

Hydrogen spillover is an interfacial phenomenon in which activated hydrogen species (H*), generated through H* adsorption on a metal surface, migrate onto the support surface, and participate in the subsequent reaction step. Originally identified in thermocatalytic hydrogenation, where supports with strongly negative ΔG_H*_ render H* migration thermodynamically spontaneous, this phenomenon has more recently been recognized as a significant mechanistic pathway in electrocatalytic HER [24,25].

In the electrocatalytic HER, hydrogen spillover provides an alternative design strategy beyond conventional ΔG_H*_-based optimization. A bicomponent metal/support electrocatalyst can be designed such that the metal component provides efficient H* generation, while the support offers favorable H* migration pathways and desorption sites. This configuration enables the HER without requiring a single active site to simultaneously satisfy both adsorption and desorption criteria [24].

Theoretical investigations have demonstrated that the work function difference (ΔΦ) between the metal and support is a critical parameter governing the spillover efficiency. A large ΔΦ induces interfacial charge accumulation, strengthening H* adsorption at the interface and raising the kinetic barrier for spillover. Conversely, a small ΔΦ promotes charge dilution and facilitates efficient H* migration, thereby enhancing the HER kinetics [25,26]. Furthermore, recent theoretical work has demonstrated a direct correlation between the free energy difference in hydrogen adsorption on the two components (|ΔG_H(metal)_ − ΔG_H(support)_|) and the kinetic barrier for H* transfer. Minimizing this difference by matching the d-band centers of the two components lowers the spillover barrier and promotes an efficient HER [27].

It is important to note that spillover contributes positively to the HER only when H* surface migration is kinetically facile. Where spillover is slow, an additional rate-limiting process can be introduced, impeding the overall catalytic performance. In this regard, the MSI-driven modulation of ΔΦ and ΔG_H*_ compatibility is essential for enabling efficient spillover and maximizing its contribution to HER activity (Table 2).

## 3. Design Strategies for HER Electrocatalysts

### 3.1. Metal Catalysts

#### 3.1.1. Single-Atom Catalysts

**Figure 2:**
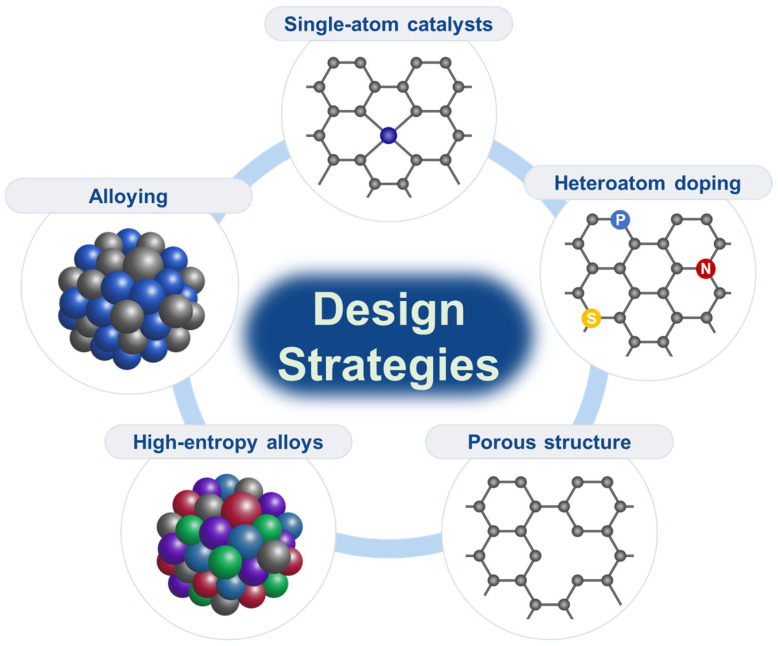
Schematic illustration of representative design strategies for HER electrocatalysts.

Single-atom catalysts (SACs) represent a class of heterogeneous catalysts in which isolated metal atoms are isolated and stabilized on a solid support, with no direct metal–metal bonding between active sites (Figure 2) [56,57]. Since their first report by Zhang and coworkers [58], SACs have attracted considerable attention as a powerful approach for bridging homogeneous and heterogeneous catalysis, owing to their well-defined active sites and tunable electronic properties.

Beyond maximizing atomic utilization, SACs exhibit high activity and selectivity during the HER owing to their unique electronic structure arising from the low-coordination environment and strong metal-support interactions [59,60]. The individually dispersed active sites allow precise tuning of the coordination environment, including the number, type, and geometry of coordinating atoms, to directly modulate ΔG_H*_ and optimize HER activity [61].

Isolated metal atoms in SACs exhibit high chemical activity due to their low-coordination environment, which significantly enhances the mass activity (MA) [7,49]. The reduced coordination number leads to unsaturated bonding states, promoting stronger interactions with reactant intermediates and facilitating more favorable H* adsorption energetics. In SAC systems, HER activity is governed by the anchoring capability of the support, the local coordination environment of metal atoms, and the synergistic interactions with neighboring metal atoms or nanoclusters.

Yin et al. demonstrated that the catalytic performance of Pt SACs is highly sensitive to the coordination environment of isolated Pt atoms by preparing two structurally distinct single-atom catalysts anchored on graphdiyne (GDY) [35]. Pt atoms coordinated with alkynyl C atoms formed either five-coordinated C_1_-Pt-Cl_4_ species (Pt-GDY1) or four-coordinated C_2_-Pt-Cl_2_ species (Pt-GDY2), and this subtle difference in coordination geometry led to a significant divergence in catalytic behavior. Pt-GDY2, with its lower coordination number, exhibited a higher unoccupied density of states of the Pt 5d orbital and near-zero ΔG_H*_, collectively contributing to superior HER activity. This work provides direct evidence that precise engineering of the coordination environment is an effective approach for optimizing the intrinsic activity of Pt single-atom sites.

Kuang et al. demonstrated that Pt single atoms supported on N-doped mesoporous hollow carbon spheres (Pt_1_/NMHCS) achieve outstanding HER activity through precise control of the coordination environment of isolated Pt atoms [36]. The isolated state of Pt_1_ single atoms, stabilized through the synergistic combination of N doping and a 3D hollow structure, generates highly positively charged Pt_1_ with an increased density of unoccupied 5d-electron states, which is directly responsible for the enhanced intrinsic site activity. Pt_1_/NMHCS delivered superior HER performance relative to both N-free Pt_NP_/MHCS and commercial Pt/C (Pt: 20 wt.%), confirming the advantage of single-atom dispersion over nanoparticle counterparts. Notably, the isolated Pt single atoms maintained their atomic dispersion after accelerated durability tests, whereas Pt nanoparticles underwent agglomeration. This highlights the pivotal role of strong metal-support interactions in preserving the single-atom structure during prolonged electrochemical operation.

**Figure 3:**
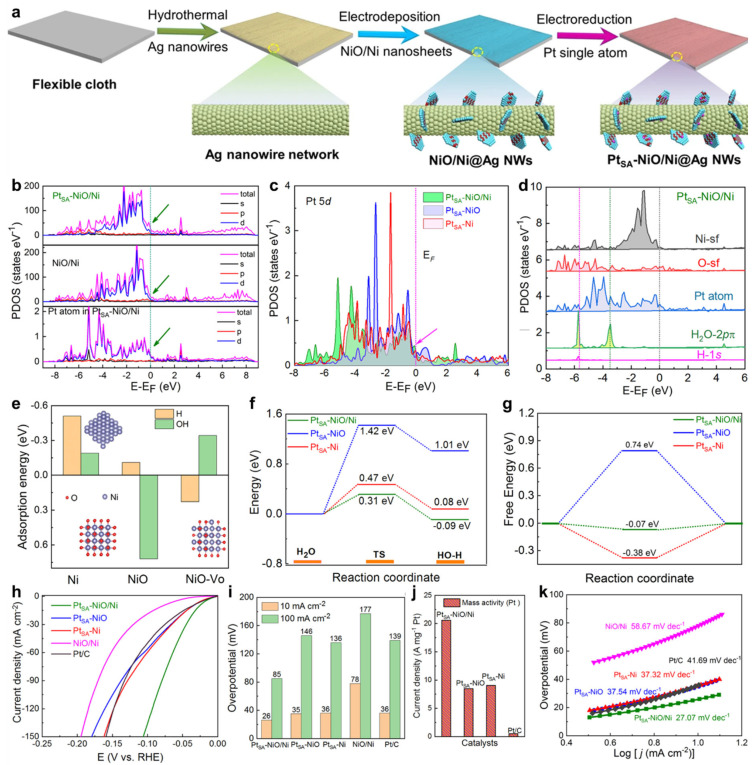
(**a**) Schematic illustration of synthesis and water splitting mechanism of Pt_SA_-NiO/Ni. (**b**) Calculated PDOS of NiO/Ni and Pt_SA_-NiO/Ni, with aligned Fermi level. (**c**) Calculated Pt 5*d* band of Pt_SA_-NiO/Ni, Pt_SA_-NiO, and Pt_SA_-Ni. (**d**) The orbital alignment of the surficial sites for Pt_SA_-NiO/Ni binding with H_2_O molecule. (**e**) Calculated OH-binding energies (Δ*E*_OH_) and H binding energies (Δ*E*_H_) for Ni, pure NiO, and O vacancies-modified NiO surface. (**f**) Calculated energy barriers of water dissociation kinetic and (**g**) Adsorption free energies of H*. (**h**) HER polarization curves. (**i**) Comparison of overpotentials. (**j**) Mass activity. (**k**) Corresponding Tafel slope originated from LSV curves. Adapted with permission from Ref. [4].

Zhou et al. developed a single-atom Pt (Pt_SA_) anchored NiO/Ni heterostructure nanosheet on an Ag nanowire (Ag NW) network nanocomposite (Pt_SA_-NiO/Ni) via a facile electrodeposition strategy (Figure 3) [4]. Immobilizing Pt single atoms at the NiO/Ni interface elevated the occupation of the Pt 5d orbital at the Fermi level, generating more free electrons on Pt sites and yielding a near-optimal ΔG_H*_ value of −0.07 eV, compared to −0.38 eV on Ni and 0.74 eV on NiO. This optimized hydrogen binding energy effectively promoted the conversion of H* and desorption of H_2_, resulting in enhanced alkaline HER performance.

#### 3.1.2. Alloying

Alloying is one of the most widely employed strategies to enhance the HER performance of Pt-based catalysts while simultaneously reducing noble metal usage [62]. Upon alloy formation, electrons are redistributed around the constituent atoms due to differences in their chemical properties, modulating the d-band center and thereby lowering the energy barriers associated with intermediate adsorption at active sites [6,63]. The formation of alloys optimizes the hydrogen adsorption energy through three cooperative mechanisms.

The ligand effect arises from an electronegativity-driven charge redistribution between the host and neighboring foreign metal atoms, which shifts the d-band center [64,65]. The strain effect originates from lattice compression or expansion due to differences in atomic radii among the constituent metals, altering the d-band structure [66]. The ensemble effect refers to the emergence of new active site configurations upon alloying, enabling reaction pathways inaccessible on pure metal surfaces [67]. These combined effects collectively shift ΔG_H*_ toward the thermoneutral optimum, enabling Pt-M alloys to simultaneously reduce the material cost while improving the catalytic activity and cyclic stability [68,69].

Yang et al. confined Pt single atoms within a nitrogen-doped porous carbon matrix alongside CoPt alloy nanocrystals (CoPt-Pt_SA_/NDPCF), in which the CoPt alloy plays a central role in regulating the electronic properties of the adjacent Pt single atoms [49]. DFT calculations revealed that the CoPt alloy modulates the d-band occupancy near the Fermi level, generating more free electrons to enhance the conversion of H*. As a result, CoPt-Pt_SA_/NDPCF with a low Pt loading of (0.42 wt.% exhibited superior HER performance at a high current density compared to commercial Pt/C (Pt: 10 wt.%) under both acidic and alkaline conditions, along with excellent long-term stability enabled by the protective encapsulated structure.

Kuang et al. demonstrated that intermetallic Pt_3_Fe alloy nanoparticles supported on activated N-doped mesoporous carbon spheres (Pt_3_Fe/NMCS-A) achieve pH-universal HER activity through the alloy-induced modulation of the electronic structure [9]. DFT calculations revealed that alloying Fe with Pt lowers the ε_d_ value of the Pt 5d orbital, reducing the H* adsorption strength and yielding a near-optimal |ΔG_H*_| for an acidic HER. Furthermore, the bifunctional nature of the Pt_3_Fe alloy, in which Pt and Fe serve as co-adsorption sites for H* and OH* intermediates, respectively, facilitates water dissociation under alkaline and neutral conditions, overcoming the key kinetic barrier of the alkaline HER. As a result, Pt_3_Fe/NMCS-A exhibited low overpotentials of 13, 29, and 48 mV at η_10_ in 0.5 M aq. H_2_SO_4_, 1.0 M KOH, and 1.0 M PBS, respectively, along with robust stability across all pH conditions.

**Figure 4:**
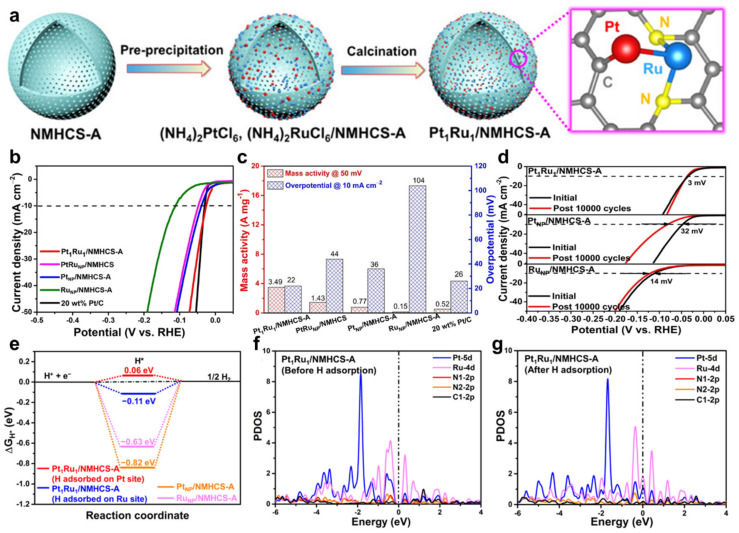
(**a**) Schematic illustration for the synthetic process of Pt_1_Ru_1_/NMHCS-A. (**b**) LSV curves of all samples in 0.5 M aq. H_2_SO_4_. (**c**) Corresponding mass activity and η_10_. (**d**) LSV curves before and after ADTs. (**e**) Calculated Δ*G*_H*_ values of different structures. Δρ and PDOS (**f**) before and (**g**) after adsorption of H on the Pt atom in the Pt_1_Ru_1_ dimer. Adapted with permission from Ref. [39].

Zhao et al. constructed a dual-atom catalyst composed of Pt-Ru dimers (Pt_1_Ru_1_) anchored on activated N-doped mesoporous hollow carbon spheres (Pt_1_Ru_1_/NMHCS-A) (Figure 4), in which the Pt-Ru dimer plays a crucial role in regulating the electronic structure of the Pt active site through strong metal–metal and metal–support interactions [39]. Spectroscopic analyses and DFT calculations indicated that the formed C_1_-Pt-Ru-N_2_ coordination structure induces the redistribution of electrons from Ru to Pt with reinforced interaction between the Pt_1_Ru_1_ dimer and the NMHCS-A support. In particular, free electrons are transferred from the Ru atom to the Pt atom through the Pt-Ru bond, while the hybridization between the Pt 5d orbital and the H 1s orbital lowers the energy level near the Fermi level, thereby enhancing electron capture and proton reduction capabilities. Pt_1_Ru_1_/NMHCS-A exhibited significantly enhanced HER activity through the synergistic effect of the Pt-Ru dual-atom structure, providing new insights into rational designs of highly efficient dual-atom catalysts for the HER.

#### 3.1.3. High-Entropy Alloys

While binary and ternary alloys offer effective tuning of HBE, alloying of multicomponents is still highly challenging due to phase segregation driven by the differing miscibility of elements. HEAs are single-phase alloys composed of at least five different elements, with each element contributing between 5% and 35% of the atomic composition [70,71]. HEAs have recently emerged as a promising strategy, offering superior catalytic activity and stability even with a low Pt content through synergistic effects among multiple constituent elements [72]. HEA catalysts, possessing a unique and compelling set of characteristics, have exhibited significant potential for practical applications. High-entropy alloys have recently attracted much attention in the catalysis community as a promising strategy for fine-tuning the hydrogen binding energy (HBE) via multicomponent design approaches. This is attributed to their four core effects of high entropy, lattice distortion, sluggish diffusion, and cocktail effects, which can substantially improve the catalytic activity and stability.

Wang et al. fabricated Ru-doped PtFeNiCuW HEA octahedral nanocrystals supported on carbon nanotubes (Ru-PtFeNiCuW/CNTs), in which a trace amount of Ru played a crucial role in promoting the homogeneous reduction and growth of multi-metallic HEA nanocrystals [40]. The incorporation of Ru facilitated the concurrent reduction of metal precursors, enabling the formation of uniform octahedral HEA nanocrystals with well-defined (111) facets despite the ultralow Ru content (<1%). Theoretical calculations revealed that W sites accelerated H_2_O adsorption and dissociation to generate abundant H* intermediates, while neighboring hollow Cu-Cu-Cu and Cu-Cu-Pt sites promoted efficient H* desorption, resulting in enhanced HER kinetics through a multi-site synergistic effect. Consequently, the Ru-PtFeNiCuW/CNTs catalyst exhibited enhanced intrinsic HER activity by integrating distinct functional active sites within the HEA structure, demonstrating the potential of compositionally complex HEAs as efficient electrocatalysts for the HER.

Feng et al. designed ultrasmall NiCoMoPtRu high-entropy alloy nanoclusters (HEANCs) with an average size of only seven atomic layers [53]. The high-entropy stabilization effect improved the structural stability and corrosion resistance of the nanoclusters, while in situ growth on carbon supports suppressed nanoparticle agglomeration during the HER. DFT calculations revealed that the optimized electronic structure of the HEANCs facilitated H_2_O adsorption and dissociation and reduced the |ΔG_H*_| values compared to pure Pt. Specifically, the Pt-5d orbital acted as an electron reservoir, whereas the Ni-3d and Co-3d orbitals near the Fermi level promoted charge redistribution during the HER. This synergistic interaction among the multicomponent active sites accelerates the Volmer step, thereby enhancing both the HER activity and stability of the HEANCs.

Wan et al. demonstrated the potential of high-entropy alloys (HEAs) as a promising platform for HER electrocatalysis by synthesizing a series of HEA@HCS composites with systematically varied compositions, ranging from quaternary PtFeCoNi to quinary and senary systems [51]. The high configurational entropy of HEA materials promotes the homogeneous distribution of multiple active sites, while electronegativity differences among the constituent elements govern the local electronic configuration to optimize hydrogen adsorption and desorption. DFT calculations revealed that the sequential introduction of Cu and Cr into PtFeCoNi@HCS optimizes the local electronic structure, giving rise to a multi-site synergistic effect that collectively enhances both HER activity and durability. This work highlights the unique ability of HEAs to simultaneously incorporate multiple functional elements, offering a versatile compositional design strategy beyond conventional binary or ternary alloy systems.

**Figure 5:**
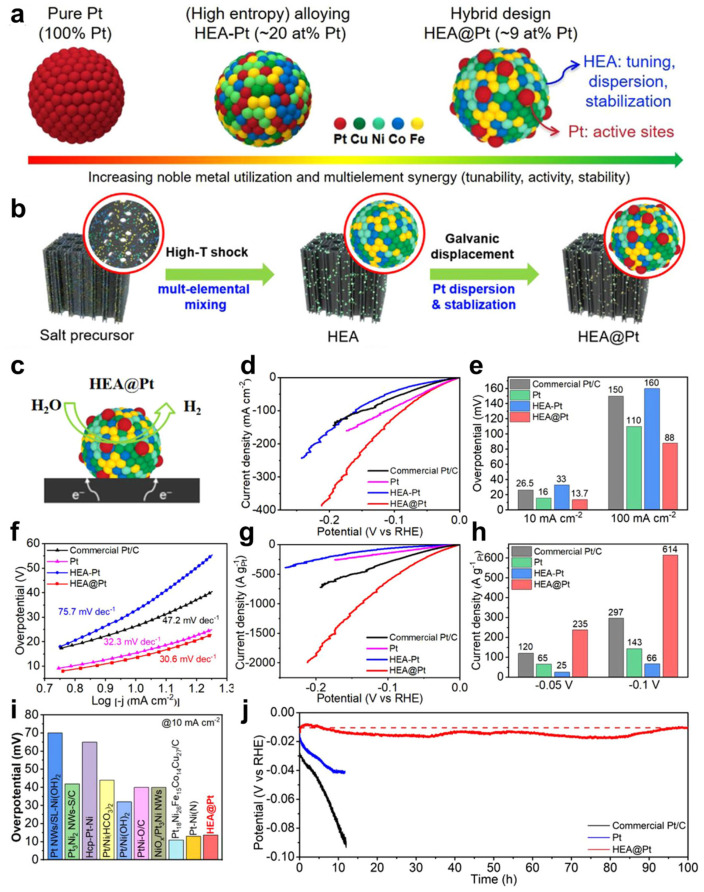
(**a**) Structure schematic images of Pt, homogeneous HEA-Pt, and HEA@Pt. (**b**) Schematic for the preparation process of HEA@Pt. (**c**) Schematic image of HEA@Pt on CW as HER catalysts. (**d**) Areal activity LSV curves. (**e**) Overpotential. (**f**) Tafel slopes. (**g**) Mass activity of LSV curves. (**h**) Mass activities. (**i**) Overpotential of HEA@Pt at 10 mA/cm^2^, compared with other reported Pt-based catalysts. (**j**) Stability tests. Adapted with permission from Ref. [55].

Shi et al. developed HEA@Pt electrocatalyst by anchoring Pt onto FeCoNiCu-based HEA nanoparticles via galvanic displacement after rapid thermal shock treatment (Figure 5) [55]. The multielement framework tuned the electronic environment of Pt, leading to enhanced hydrogen adsorption/desorption behavior and improved catalytic efficiency toward HER. Simultaneously, the entropy-driven stabilization effect enabled uniform dispersion of ultrafine Pt clusters while preventing particle coalescence under electrochemical conditions. In addition, the strong interfacial bonding between the HEA nanoparticles and the substrate, promoted by high-temperature synthesis, further improved long-term catalytic durability. Owing to these synergistic features, the HEA@Pt delivered higher activity and durability than commercial Pt/C and single-phase HEA-Pt counterparts.

### 3.2. Carbon Support

#### 3.2.1. Heteroatom Doping

In Pt-based HER electrocatalysis, carbon supports play a pivotal role not merely as an inert scaffold but as an active participant in determining catalytic performance outcomes. Heteroatom doping of the carbon support through the incorporation of N, S, B, or P into the carbon framework has emerged as a powerful strategy capable of simultaneously enhancing the intrinsic HER activity of the support, strengthening MSI, improving Pt dispersion, and tailoring the electronic structure of supported Pt species. This section discusses the mechanistic basis of heteroatom doping and its role in supporting Pt-based HER catalysts.

Heteroatom-doped carbon nanomaterials have emerged as promising electrocatalysts for the HER owing to their earth-abundance, tunable molecular architecture, excellent electrical conductivity, and remarkable tolerance to both acidic and alkaline environments [73]. The incorporation of heteroatoms into the carbon framework induces a significant charge redistribution and modulates the local electronic structure of adjacent carbon atoms, thereby generating catalytically active sites and optimizing the adsorption the ΔG_H*_ [74,75].

The introduction of heteroatoms with electronegativity either higher (e.g., N) or lower (e.g., B) than carbon breaks the charge neutrality of the carbon lattice, creating positively charged carbon sites (C^+^) that serve as preferential adsorption sites for reaction intermediates including H* [76]. This doping-induced charge asymmetry effectively lowers the reaction energy barrier and increases the density of active sites, thereby enhancing the HER kinetics [75].

While single-element doping has demonstrated considerable promise, its capacity to optimize multiple catalytic descriptors simultaneously remains limited. Co-doping strategies, in which two or more heteroatoms are introduced into the carbon framework, have been proposed as a more effective approach to exploit synergistic electronic effects [73]. Multi-heteroatom doping enables the simultaneous tuning of the electron density and the redistribution of the charge within the porous carbon matrix, providing a greater number of favorable H* adsorption sites and ultimately enhancing the electrocatalytic activity [76].

Lee et al. demonstrated that heteroatom doping of the carbon support plays a critical role in enhancing HER performance capabilities by preparing Pt nanoparticles supported on triazine graphitic nanoplatelets (Pt@TGNP) [30]. The incorporation of triazine units into the graphitic framework introduces nitrogen-containing active sites that not only serve as anchoring sites for a uniform and stable distribution of Pt nanoparticles but also modulate the local electronic structure of the support. The high electrical conductivity and mechanical stability of TGNP, preserved through its hexagonal graphene structure, further contribute to efficient charge transfer and long-term structural integrity during the HER.

Yan et al. reported that a sulfur-doped mesoporous carbon (S-C) support plays a crucial role in controlling the electronic structure of Pt atoms and the corresponding HER performance [33]. Based on spectroscopic characterizations and DFT calculations, they demonstrated that the S-C support captures electrons from Pt single atoms via the strong chemical Pt-S interaction. However, when the Pt size is increased to nanoclusters, the charge transfer direction is reversed from S-C to Pt. These results indicate that the electronic metal-support interaction (EMSI) between Pt and the S-C support can be manipulated through modification of Pt morphologies from single atoms to nanoclusters, highlighting the pivotal role of the S-doped carbon support in tuning the charge transfer behavior and HER performance.

Zhang et al. developed an anchoring-site engineering strategy to construct ultrafine PtMo alloy nanocrystals supported on N-doped carbon (PtMo-NC) for HER electrocatalysis [54]. The abundant nitrogen sites in the carbon support effectively anchored metal ions and suppressed excessive aggregation during pyrolysis, facilitating the formation of ultrafine PtMo nanocrystals. In addition, the porous structure of the N-doped carbon support promoted efficient electrolyte transport during the HER. The strong interaction between the nitrogen sites and PtMo nanocrystals further enhanced the structural stability of the catalyst and prevented nanoparticle overgrowth during the heat treatment.

**Figure 6:**
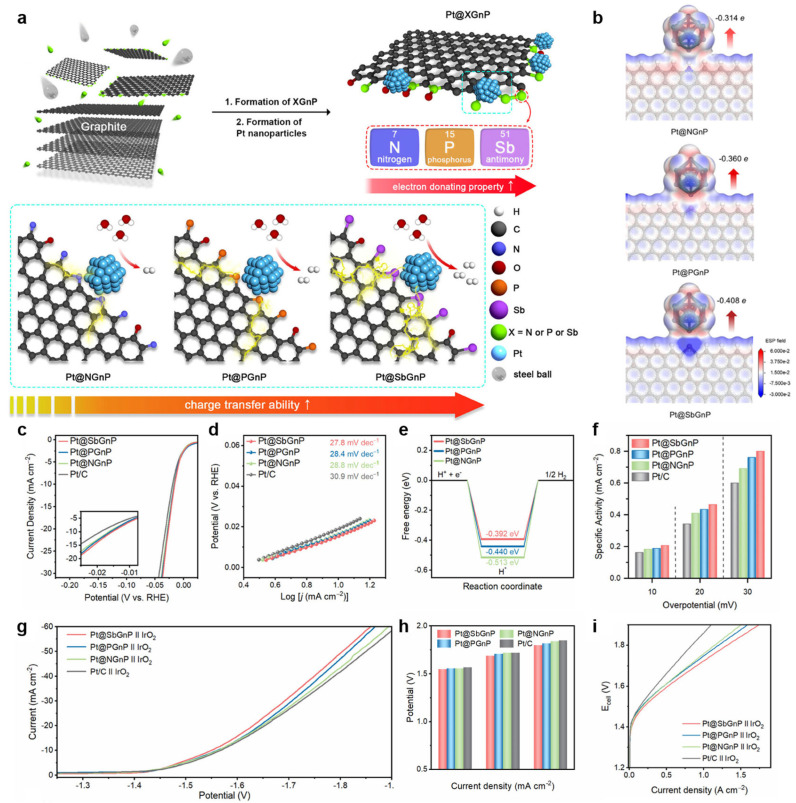
(**a**) Schematic representation of the synthesis process for Pt@XGnPs (X = N, P, or Sb). (**b**) Electrostatic potential maps around of the optimized Pt@XGnP. Half-cell electrocatalytic performance of Pt@XGnPs: (**c**) HER polarization curves, (**d**) Tafel plots, (**e**) H-adsorption free energy diagram, and (**f**) mass activities of electrocatalysts. Overall water-splitting system of Pt@XGnPs: (**g**) Polarization curves and (**h**) Comparison of current density levels. PEMWE performance of Pt@XGnPs: (**i**) polarization curves. Adapted with permission from Ref. [32].

Baek et al. investigated how the choice of dopant heteroatom influences metal-support interactions by preparing Pt nanoparticles supported on graphitic nanoplatelets doped with group VA elements (N, P, or Sb) (Figure 6) [32]. As the atomic period of the dopant heteroatom increased, the charge transfer to Pt nanoparticles was progressively enhanced through stronger Pt-X bonding. This led to more pronounced electronic modification of the supported Pt species, directly influencing the catalytic activity of Pt. In particular, Sb doping proved most effective owing to its high electropositivity and larger atomic radius. These properties maximized the charge transfer to Pt and facilitated an optimal Pt dispersion, ultimately boosting the electrochemical surface area through enhanced EMSI. This work establishes heteroatom periodicity as a key design parameter for tuning MSI and optimizing the catalytic performance of heteroatom-doped carbon-supported Pt catalysts.

#### 3.2.2. Porous Structure

Porous carbon substrates have emerged as one of the most effective support platforms for HER electrocatalysts owing to their high specific surface area, tunable pore architecture, and excellent electrical conductivity [76]. The high surface area promotes the uniform dispersion of catalytically active sites, while the interconnected porous framework provides continuous channels for electrolyte diffusion and an efficient release of evolved H_2_ gas, thereby minimizing the mass transport resistance during the HER process [77,78,79].

Beyond their structural role, porous carbon supports actively modulate the catalytic behavior of supported metal species through intrinsic structural defects. Vacancy and topological defects within the carbon matrix function as anchoring sites for metal nanoparticles and promote electron transfers between the metal species and the support, directly tuning the electronic structure of the active sites and reducing the energy barrier for hydrogen adsorption and desorption [80].

Optimizing the balance between micropores and mesopores is central to maximizing the HER performance. Micropores increase the density of exposed active sites, whereas mesopores serve as open transport channels that facilitate reactant access and product removal. Achieving this dual functionality through controlled synthesis represents the key design principle for porous-carbon-supported HER electrocatalysts [81].

Zeng et al. synthesized highly dispersed Pt entities supported on mesoporous N-doped carbon (MPNC) nanospheres for enhanced HER performance [41]. The continuous mesoporous structure and high surface area of the MPNC support effectively suppressed Pt atom diffusion and aggregation during catalyst synthesis, facilitating the formation of highly dispersed Pt single atoms, clusters, and nanoparticles through a mesopore-confinement effect. In addition, the nitrogen functionalities of the MPNC support strongly interacted with Pt species, enabling the anchoring and stabilization of Pt single atoms on the carbon surface. Pt/MPNC exhibited significantly higher HER geometric and Pt mass activities than commercial Pt/C and Pt/CNF catalysts, driven by its large electrochemically active surface area and high density of well-dispersed Pt active sites on the mesoporous N-doped carbon support.

Li et al. developed a low-Pt-content electrocatalyst by depositing Pt nanoparticles onto a three-dimensional tungsten oxide/reduced graphene oxide aerogel (WGA) [42]. The interconnected porous structure of the WGA support promoted mass transport and electron transfers while providing a large exposed surface area for Pt active sites. In addition, oxygen vacancies introduced into the WO_3-x_ framework induced electron transfers from WO_3-x_ to Pt nanoparticles, which optimized the electronic structure of Pt, downshifted the d-band center, and reduced the hydrogen adsorption energy. The strong metal-support interaction further stabilized the Pt nanoparticles and suppressed their aggregation during the HER. Moreover, the hydrogen spillover effect facilitated the migration of adsorbed H* species, accelerating the overall HER kinetics. Accordingly, the Pt/WGA catalyst with only 0.8 wt.% Pt showed enhanced HER activity and stability due to the synergistic effects of the porous aerogel structure and the Pt-WO_3-x_ electronic interaction.

Kim et al. reported Pt nanoparticles supported on activated phosphorus-doped graphene (A_PGnP) to enhance the HER performance [31]. The incorporation of phosphorus (P) atoms improves the catalytic performance by modulating the electronic structure between the Pt and P within the carbon matrix. A_PGnP, endowed with abundant active sites, a large surface area, and P doping, improves the performance of the HER under acidic conditions. As a result, Pt/A_PGnP with a lower Pt loading rate (12.75 wt.%) exhibited a comparable overpotential and reduced Tafel slopes while showing superior stability relative to that of commercial Pt/C (Pt: 20 wt.%). Therefore, Pt/A_PGnP exhibited high activity and stability, indicating its potential as a stable HER catalyst.

**Figure 7:**
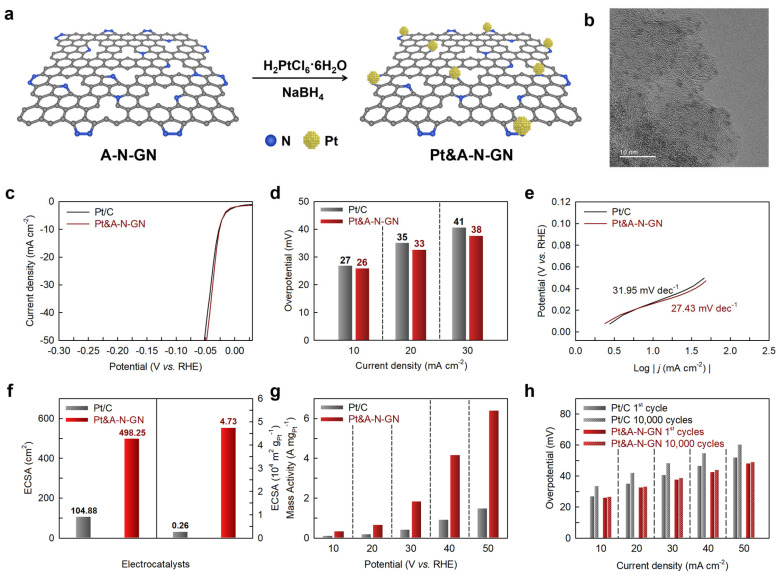
(**a**) Schematic illustration of Pt&A-N-GN synthesis. (**b**) HR-TEM images of Pt&A-N-GN. Electrocatalytic HER performance of Pt/C and Pt&A-N-GN under acidic media: (**c**) Polarization curves, (**d**) Overpotential values, (**e**) Corresponding Tafel slopes, (**f**) Electrochemical active surface area (ECSA), (**g**) Mass activity value at a specific overpotential, and (**h**) Overpotential changes at different current densities before and after 10,000 cycles. Adapted with permission from Ref. [28].

Yoon et al. demonstrated that the porous structure of the carbon support plays a pivotal role in enhancing HER performance capabilities by loading Pt nanoparticles onto activated nitrogen-doped graphitic nanoplatelets (A-N-GN) (Figure 7) [28]. The activation process generated additional pores and enlarged the surface area, providing more accessible active sites and improving the mass transport efficiency. As a result, Pt&-N-GN showed superior HER performance in acidic media, surpassing that of commercial Pt/C (Pt: 20 wt.%). These results indicate that rational engineering of the porous structure of the carbon support is a promising strategy for maximizing the utilization of supported Pt nanoparticles and enhancing the overall electrocatalytic activity.

## 4. Conclusions and Perspectives

In this review, we have discussed rational design strategies for Pt-based HER electrocatalysts, with a focus on metal-level engineering and support-level engineering as two complementary and synergistic approaches to maximizing catalytic performance outcomes while minimizing Pt usage levels.

At the metal level, SACs have demonstrated exceptional atomic utilization efficiency and tunable HER activity through precise control of the local coordination environment and strong metal-support interactions. Alloying strategies, including those that rely on binary alloys, intermetallic compounds, and HEAs, enhance the HER activity by tuning the d-band center through synergistic electronic effects. This modulation optimizes hydrogen adsorption/desorption and accelerates water dissociation during the HER. Despite these advantages, several limitations remain. SACs are still associated with limited long-term stability due to atom migration and aggregation under practical operating conditions. Meanwhile, alloy and HEA systems often exhibit structural complexity and heterogeneous active sites, making precise control of the electronic interactions among multimetallic components difficult. In addition, achieving both high intrinsic activity and long-term durability across a wide pH range remains challenging through metal engineering alone.

To overcome these limitations, increasing attention has been directed toward carbon-support engineering strategies. Heteroatom doping, such as N, P, S, or B incorporation into carbon frameworks, can effectively regulate the electronic structure of Pt-based active sites through charge redistributions and strengthened metal-support interactions. Simultaneously, porous structure engineering provides large surface areas, abundant anchoring sites, and interconnected transport pathways for electrons and electrolytes. These structural advantages suppress nanoparticle aggregation, increase active-site exposure, and facilitate mass and charge transport during the HER. Importantly, the synergistic combination of heteroatom doping and porous carbon architectures enables both electronic and structural optimization of electrocatalysts, offering advantages that cannot be achieved by metal design approaches alone.

Therefore, the rational integration of advanced metal engineering strategies with heteroatom-doped porous carbon supports represents a promising pathway toward highly active, durable, and low-Pt HER electrocatalysts. Looking forward, further advances in operando characterization techniques and computational approaches, including machine-learning-assisted catalyst design, are expected to accelerate our understanding of metal-support interactions and catalytic descriptors. By combining large experimental and computational datasets with high-throughput screening and predictive modeling, machine learning can facilitate the identification of key catalytic descriptors, optimization of Pt utilization, and rational design of catalyst architectures. Continued efforts that elucidate the synergistic mechanisms between active metals and engineered carbon supports will be essential for the development of practical and sustainable HER electrocatalysts for green hydrogen production.

## Figures and Tables

**Table 1:** HER Mechanism in Acidic and Alkaline Conditions.

Step	Acidic	Alkaline
Volmer	H^+^ + e^−^ + M → M-H_ads_ + H_2_O	H_2_O + e^−^ + M → M-H_ads_ + OH^−^
Heyrovsky	M-H_ads_ + H_3_O + e^−^ → H_2_ + H_2_O + M	M-H_ads_ + H_2_O + e^−^ → H_2_ + OH^−^ + M
Tafel	2M-H_ads_ → H_2_ + 2M	

**Table 2:** Electrocatalytic Properties of Pt-based HER Catalysts.

Metal	Support	Overpotential (mV) (at 10 mA/cm^2^)	Tafel Slope (mV/dec)	Electrolyte	Ref.
Pt	A-N-GN	26	27.43	0.5 M H_2_SO_4_	[28]
Pt	SbGnP	13	28.8	0.5 M H_2_SO_4_	[29]
Pt	TGNP	32	28.44	0.5 M H_2_SO_4_	[30]
Pt	A_PGNP	31	31.09	0.5 M H_2_SO_4_	[31]
Pt	SbGnP	15.3	27.8	0.5 M H_2_SO_4_	[32]
Pt	PGnP	16.0	28.4	0.5 M H_2_SO_4_	
Pt	NGnP	16.5	28.8	0.5 M H_2_SO_4_	
Pt	S-C	11	23.51	0.5 M H_2_SO_4_	[33]
Pt	WS_2_	31	27.2	0.5 M H_2_SO_4_	[34]
PtSA	GDY2	50	46.6	0.5 M H_2_SO_4_	[35]
Pt_1_	NMHCS	40	56	0.5 M H_2_SO_4_	[36]
0.22Pt	HMoS_2_	44	34.83	0.5 M H_2_SO_4_	[37]
0.34Pt	HMoS_2_	43	35.84	0.5 M H_2_SO_4_	
PtNi_NPs_	Au_SA_-NDC	19.1	21	0.5 M H_2_SO_4_	[38]
Pt3Fe	NMCS-A	13	21	0.5 M H_2_SO_4_	[9]
Pt_1_Ru_1_	NMHCS-A	22	38	0.5 M H_2_SO_4_	[39]
Ru-PtFeNiCuW	CNT	9	19.2	0.5 M H_2_SO_4_	[40]
Pt	MPNC	12	-	0.5 M H_2_SO_4_	[41]
Pt	WGA	42	30	0.5 M H_2_SO_4_	[42]
Pt	MgO	39	39	1 M KOH	[43]
Pt	D-NiFe-LDH	4	52.71	1 M KOH	[44]
Pt-SA	FNS	77	68	1 M KOH	[45]
Pt-NP	FNS	42	79	1 M KOH	
PtSA-PtC	NDPCM	14	58.9	1 M KOH	[46]
PtRu	WO_3_-O_V_	9	25.4	1 M KOH	[47]
Pt	α-MoC	26	23	1 M KOH	[48]
CoPt	NDPCF	31	43.65	1 M KOH	[49]
Pt/CeO_x_	C	19	50	1 M KOH	[50]
PtSA-NiO	Ni	26	27.07	1 M KOH	[4]
PtFeCONi	HCS	89	157.31	1 M KOH	[51]
PtFeCONiCu	HCS	62	136.42	1 M KOH	
Dr-Pt	CNT	26	52	1 M KOH	[52]
Dp-Pt	CNT	45	71	1 M KOH	
HEANC	C	9.5	29.8	1 M KOH	[53]
PtMo	NC	47	32	1 M KOH	[54]
HEA@Pt	CW	13.7	30.6	1 M KOH	[55]

## Data Availability

Data presented in this study are available on request from the corresponding authors.
